# Silk Sericin-Based Electrospun Nanofibers Forming Films for Cosmetic Applications: Preparation, Characterization, and Efficacy Evaluation

**DOI:** 10.3390/molecules30030715

**Published:** 2025-02-05

**Authors:** Ivana Dragojlov, Rony Aad, Diletta Ami, Marco Mangiagalli, Antonino Natalello, Simone Vesentini

**Affiliations:** 1 Department of Electronics, Information, and Bioengineering, Politecnico di Milano, 20133 Milan, Italy; ivana.dragojlov@polimi.it (I.D.); rony.aad@polimi.it (R.A.); 2 Department of Biotechnology and Biosciences, University of Milano-Bicocca, 20126 Milano, Italy; diletta.ami@unimib.it (D.A.); marco.mangiagalli@unimib.it (M.M.); antonino.natalello@unimib.it (A.N.)

**Keywords:** silk-sericin, degumming, electrospinning, eye patches, sericin-based films, moisturizing, elasticizing, smoothing

## Abstract

This study investigates the extraction, characterization, and cosmetic application of silk sericin, a protein derived from *Bombyx mori* silkworm cocoons, with a focus on its potential in sustainable and biodegradable cosmetic formulations. Sericin was extracted using a high-temperature, high-pressure autoclave degumming method and spray-dried into a stable powder. The molecular weight distribution of sericin was analyzed, revealing fractions ranging from 10 to 37 kDa in Elution 1A and 25–40 kDa in Elution 1B. Electrospinning of sericin led to increased β-sheet content compared to raw sericin, as shown by secondary structure analyses. The electrospun sericin was then blended with gelatin to enhance mechanical strength and stability, resulting in robust films suitable for cosmetic applications. These films were developed into eye contour patches designed to deliver moisturizing, elasticizing, and smoothing effects. The efficacy of the patches was evaluated in 20 participants, showing increased skin elasticity (+35.1%) and smoothness (R_a_: −30.7%, R_z_: −26.6%), though a decline in hydration was observed, potentially indicating opportunities for further optimization.

## 1. Introduction

Sericin accounts for 25–30% of the silk protein produced by *Bombyx mori* silkworms, alongside fibroin [1]. It serves as an adhesive holding the fibroin fibers together [2]. Traditionally regarded as a by-product of the silk industry, sericin is released during the degumming process, with the water containing it often discarded. However, recent interest in sericin has grown due to its diverse biophysical properties, positioning it as a promising material for a wide range of applications [1]. These properties include biocompatibility, biodegradability, anti-inflammatory, antioxidant, antibacterial, UV protection, anti-aging, anti-tyrosinase, and anti-cancer effects [1]. As a result, sericin has found use in biomedical fields, such as drug delivery, tissue engineering, and serum-free cell culture media, as well as in textiles, cosmetics, and food packaging [1,2].

Sericin is a hydrophilic protein with a random coil and β-sheet structure [1]. It is soluble in water at temperatures above 50 °C, forming a gel at lower temperatures [1]. Rich in hydroxyl, carboxyl groups, and polar amino acids, sericin’s amino acid profile is dominated by serine, along with 17 other amino acids [1]. Its solubility and molecular weight (ranging from 10 to 400 kDa) vary based on extraction method, temperature, pH, and processing time [3].

Extraction methods significantly influence sericin’s structure, properties, and molecular weight. High-temperature extractions typically yield molecular weights of 100–200 kDa [4], while high-temperature, high-pressure methods produce 25–150 kDa [3]. Urea-based extractions cover a broad range of 10–225 kDa, acid-based methods yield 50–150 kDa, and alkaline methods produce 15–75 kDa [3]. Enzymatic methods result in lower molecular weights, typically between 5 and 25 kDa [5]. These variations in molecular weight and structural properties impact sericin’s potential applications. Thermal extraction using hot distilled water is the most commonly employed method, offering simplicity, low environmental impact, and minimal chemical use [6].

Sericin has proven to be an effective moisture-retaining agent with excellent film-forming capabilities, offering benefits such as enhanced keratin affinity, skin care advantages, antioxidant properties, and UV protection [3].

Sericin’s soothing and anti-inflammatory effects, along with its ability to promote skin regeneration and collagen production, make it an ideal ingredient for sensitive skin and a highly sought-after addition to anti-aging skincare products [6,7].

Silk-based cosmetics, which began in the 1960s with the creation of silk cream containing fibroin, have recently shifted focus to harnessing the beneficial properties of sericin [3]. Numerous studies have since explored sericin’s role in cosmetics, highlighting its moisturizing, anti-aging, skin-soothing, and elasticizing properties, further solidifying its potential as a key ingredient in modern skincare formulations.

Sheng et al. emphasized sericin’s potential in skincare due to its similarity to collagen and its role in cosmetics. Sericin’s amino acid profile closely mirrors the natural moisturizing factor (NMF) in the skin, with a serine content of 29.34%, comparable to NMF’s 30%. Its exposed polar groups enhance hydration. A 3% sericin solution demonstrated similar moisture absorption to 60% glycerol but without clogging pores. Additionally, sericin improved hair strength, reduced tyrosine activity by over 50%, inhibited melanin production, and promoted skin whitening, showcasing its versatility in cosmetic applications [8].

Singh et al. synthesized sericin/β-cyclodextrin materials, which enhanced thermal stability, antioxidant activity, and moisture transport. The treated cotton fabrics showed excellent UV protection and skincare benefits, maintaining their properties even after multiple washes, making them promising for use in cosmetotextiles [9].

Wang et al. demonstrated that hydrothermal extraction of sericin at 220 °C resulted in high tyrosinase inhibition, which is crucial for skin whitening applications. This method offers a chemical-free, bioavailable form of sericin, making it a promising candidate for cosmetic use due to its reduced toxicity and enhanced efficacy in inhibiting melanin production [10].

Kim et al. found that sericin improves skin hydration by promoting the production of proteins that strengthen the skin’s moisture barrier, helping it retain key amino acids. This effect could be especially beneficial for dry skin or conditions like atopic dermatitis, improving overall skin hydration and appearance [11].

This study investigates the extraction and cosmetic potential of silk sericin derived from *Bombyx mori* silkworms. Sericin was isolated using a high-temperature, high-pressure autoclave method and processed into a stable powder via spray drying. Its ability to be electrospun into nanofibers was explored, and these nanofibers were combined with gelatin to create durable films used as eye contour patches. The patches are water-soluble and can be absorbed by the skin when residual water is present on its surface. Their efficacy in enhancing skin hydration, elasticity, and smoothness was evaluated through a clinical trial involving 20 participants, with pre- and post-application measurements confirming their performance.

By exploring the potential of sericin-based nanofiber films in cosmetic applications, this study not only highlights their promise in enhancing skin elasticity and smoothness but identifies critical challenges in hydration performance, paving the way for future advancements in sericin-based skincare solutions.

## 2. Results

### 2.1. Biochemical Characterization of Sericin by SDS-PAGE and Mass Spectrometry

**Figure 1:**
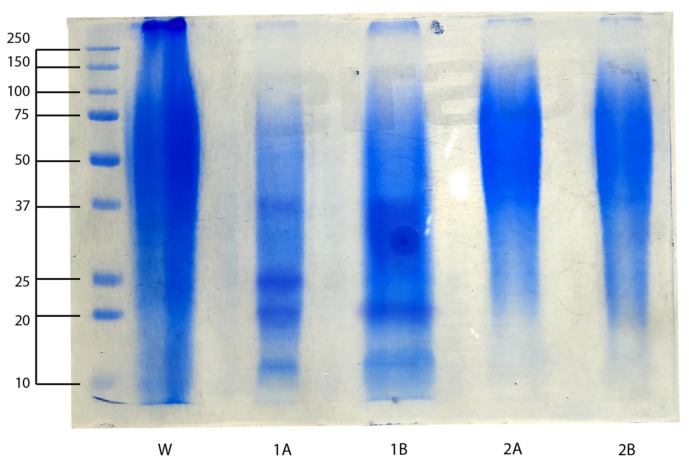
SDS-PAGE Analysis of Waste Fraction and Elution Fractions (1A, 1B, 2A, and 2B).

The molecular weight distribution of sericin, extracted using the high-temperature, high-pressure (HTHP) method, was analyzed by sodium dodecyl sulfate–polyacrylamide gel electrophoresis (SDS-PAGE), while mass spectrometry (MS) was employed to confirm its identity and assess its peptide composition. The SDS-PAGE analysis revealed distinct protein bands across specific lanes corresponding to the processed elutions (Figure 1). Clear protein bands were observed in the earlier elution fractions (1A and 1B), while no detectable bands were found in the waste or later elutions (W, 2A, and 2B), indicating effective protein isolation. The molecular weight marker, ranging from 10 to 250 kDa, facilitated the identification of molecular weight ranges.

Elution 1A exhibited prominent bands primarily between 10 and 37 kDa, with the most intense bands observed between 20 and 25 kDa, demonstrating successful enrichment of low molecular weight sericin. Compared to 1A, Elution 1B contained lower molecular weight components (~10 kDa and 20 kDa), but also a broader smear in the 25–37 kDa range, indicating a mixture of low and medium molecular weight sericin. By contrast, the lanes corresponding to 2A and 2B exhibited a much broader smear, extending from 37 to 140 kDa, suggesting the presence of medium and high molecular weight sericin fractions. This pattern may reflect differences in the elution protocol, potentially leading to protein aggregation or partial degradation during sample preparation.

Mass spectrometry analysis was conducted on tryptic peptides obtained from excised gel bands corresponding to Elutions 1A, 1B, 2A, and 2B, as shown in Figure 1. The results confirmed the predominance of Sericin 1 as the main protein component, with peptides matching its molecular weight of approximately 119.7 kDa, supported by high Mascot scores across the elutions.

Specifically, Elution 1A yielded a Mascot score of 13,067 with 31 unique peptides, 1B yielded a Mascot score of 14,432 with 34 unique peptides, 2A yielded a Mascot score of 9012 with 32 unique peptides, and 2B yielded a Mascot score of 7905 with 30 unique peptides. These results confirm the presence of Sericin 1 in all analyzed elutions and support its identification with high confidence.

Minor fractions of other proteins, including fibroin fragments, were detected in the samples but with significantly lower Mascot scores, reinforcing the specificity of sericin identification. The identified peptides exhibited minimal post-translational modifications, such as carbamidomethylation of cysteine and oxidation of methionine, which are consistent with the applied sample preparation protocols.

A comprehensive overview of the mass spectrometry results, including protein identification, peptide sequences, molecular weight distribution, and detected modifications, is provided in the Supplementary Materials (Table S1). These data offer a detailed molecular characterization of sericin, supporting its successful extraction and identification.

### 2.2. Structural Characterization by FTIR, CD, and SEC Analyses

#### 2.2.1. Structural Characterization of HTHP Sericin Powder

The secondary structures of the HTHP sericin samples were analyzed using Attenuated Total Reflection–Fourier Transform Infrared (ATR-FTIR) spectroscopy.

**Figure 2:**
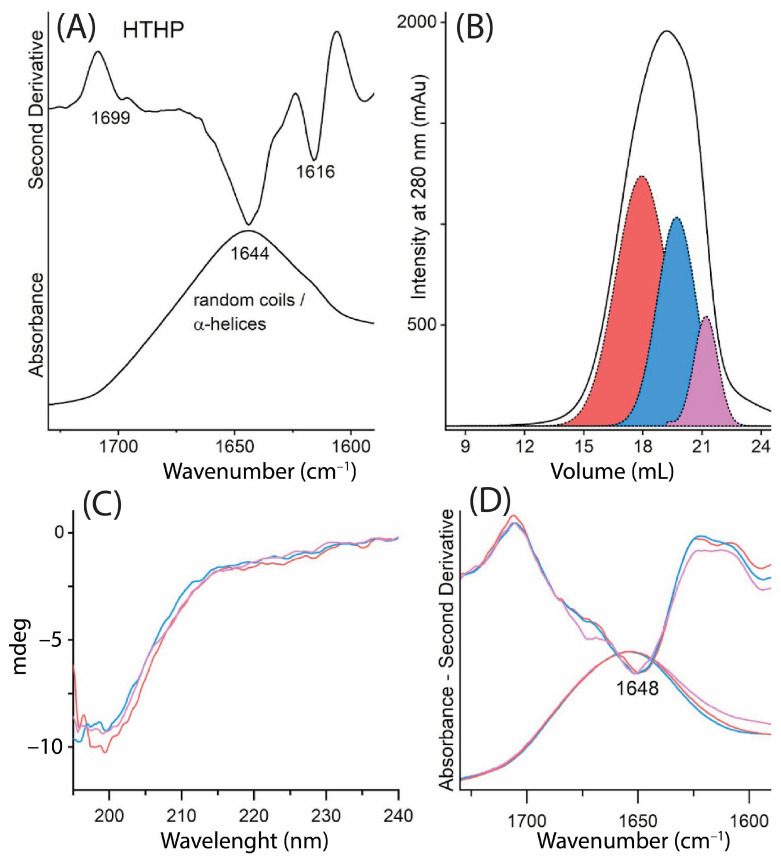
Spectroscopic characterization of the HTHP sericin powder. (**A**) FTIR absorption spectrum (upper panel) and second derivative (bottom panel) of HTHP powder in the Amide I band. Peak positions of the main components are indicated. (**B**) SEC chromatogram of HTHP sericin. The HTHP powder was resuspended in PBS (2 mg/mL), and the clarified supernatant was analyzed using a Superdex 10/200 column. Multi-Gaussian deconvolution highlights three sericin populations: HTHP SP-23 (red), HTHP SP-12 (blue), and HTHP SP-9 (magenta). The corresponding SEC fractions were collected and analyzed. (**C**) Far-UV CD spectra of the SEC fractions from panel (**B**). (**D**) FTIR absorption spectra (bottom panel) and second derivatives (upper panel) of the SEC fractions from panel (**B**). In (**C**,**D**), spectra are presented after min–max normalization for better comparison. The relatively high noise is due to suboptimal protein concentration in the SEC fractions.

In the ATR-FTIR spectrum (Figure 2A), the Amide I band was examined through both the absorbance spectrum (bottom panel) and its second derivative spectrum (upper panel). The second derivative spectrum revealed a main broad band centered at 1644 cm^–1^, which corresponds to random coil/α-helix conformations. Additionally, less intense components were observed at approximately 1616 cm^−1^ and 1699 cm^−1^, which can be attributed to β-sheet structures. These findings indicate a predominance of disordered structures, with minor contributions from β-sheets.

To complement the structural analysis and evaluate the molecular weight distribution, the HTHP sericin powder was resuspended in phosphate-buffered saline (PBS), centrifuged, and the clarified supernatant was subjected to size exclusion chromatography (SEC). Given that the molecular weight of sericin typically ranges from 10 to 400 kDa, the separation was performed using a Superdex 10/200 column (GE Healthcare, Little Chalfont, UK), with a cut-off range of 10–600 kDa [12,13]. The resulting chromatogram exhibited a broad peak with a maximum elution volume at 19.3 mL, corresponding to an estimated molecular weight of 14.7 kDa (Figure 2B, Table 1). Gaussian fitting of the chromatographic peak identified three distinct populations of sericin: HTHP SP-23 (23.8 kDa), HTHP SP-12 (12.8 kDa), and HTHP SP-9 (9.5 kDa) (Figure 2B, Table 1).

The SEC fractions corresponding to HTHP SP-23, HTHP SP-12, and HTHP SP-9 were collected and analyzed by circular dichroism (CD). The CD spectra (Figure 2C) of all SEC fractions were superimposable, showing a main minimum below 200 nm, which is characteristic of a predominantly disordered/random coil conformation. These CD results were consistent with the findings from ATR-FTIR spectroscopy (Figure 2D), where the Amide I band remained broad and centered at approximately 1648 cm^−1^, further supporting the dominance of disordered structures in the sericin fractions.

#### 2.2.2. Structural Properties of Sericin Electrospun Samples

**Figure 3:**
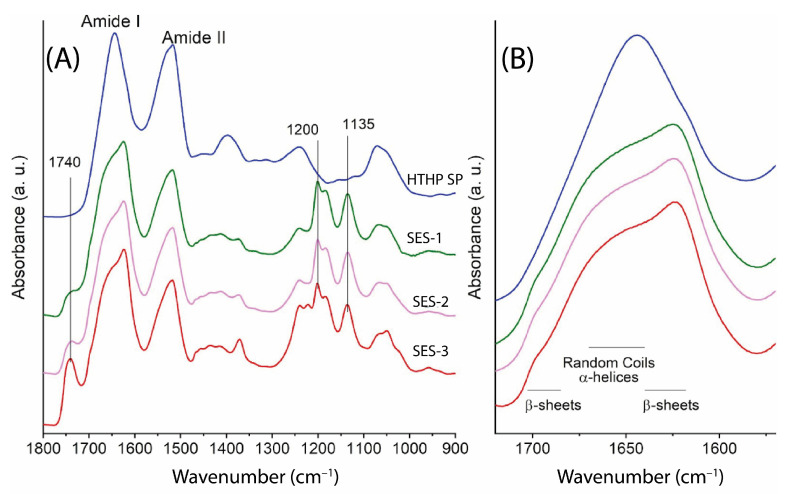
FTIR Spectra of Electrospun Sericin Samples SES-1, SES-2, and SES-3, together with HTHP Sericin Powder. (**A**) ATR-FTIR absorption spectra in the 1800–900 cm^−1^ range, highlighting the main peaks. (**B**) Spectra are reported in the Amide I band.

Sericin electrospun samples (SES-1, SES-2, and SES-3) were prepared at a concentration of 12 wt% with collector distances of 10 cm, 15 cm, and 20 cm, corresponding voltages of 17 kV, 25 kV, and 32 kV, and a constant flow rate of 1 mL/h. These samples were characterized using ATR-FTIR and scanning electron microscopy (SEM) analyses, with their ATR-FTIR spectra compared to that of the HTHP sericin powder in the 1800–900 cm^−1^ spectral range (Figure 3A). The SES samples exhibited several additional peaks that were absent in the HTHP sericin powder, which can be attributed to the absorption of trifluoroacetic acid (TFA) used during the electrospinning process [14,15]. Specifically, the peak at 1740 cm^−1^ corresponds to the C = O stretching vibration, while the peaks at 1200 cm^−1^ and 1135 cm^−1^ are associated with C–F vibrations. The ratios of C = O and C–F vibrations relative to the Amide I band area (Figure S2) enable the evaluation and comparison of residual TFA presence across the different samples. Specifically, the following trend was observed: SES-3 > SES-2 ≈ SES-1.

Although a longer collector distance (20 cm, SES-3) was expected to enhance solvent evaporation, its higher residual TFA suggests inefficient removal. Since the electric field was maintained at a constant, stretching forces remained uniform, eliminating voltage variations as a contributing factor. A denser fiber network could contribute to solvent retention; however, SEM images (see Section 2.3.1) reveal that SES-2 (15 cm) is the most compact, while SES-3 exhibits a less dense structure. This suggests that fiber packing alone does not fully explain solvent retention. Instead, localized fiber fusion in SES-3 may have restricted solvent escape, limiting post-deposition evaporation. Thus, a combination of solvent evaporation kinetics, fiber morphology, and deposition behavior likely dictated higher residual TFA retention in SES-3.

In addition, the Amide I band (Figure 3B) of the SES samples revealed two characteristic components at approximately 1621 cm^−1^ and 1698 cm^−1^, which are attributed to the presence of β-sheet structures. A broad band around 1660–1644 cm^−1^ was also observed indicating a significant contribution from disordered structures. The relative content of β-sheet structures was evaluated through curve fitting of the absorption spectra (Figure S3). The HTHP SP sample exhibited a β-sheet content of 17%, which increased to approximately 30% in the SES samples.

#### 2.2.3. Structural Properties of Sericin–Gelatin Electrospun Samples

**Figure 4:**
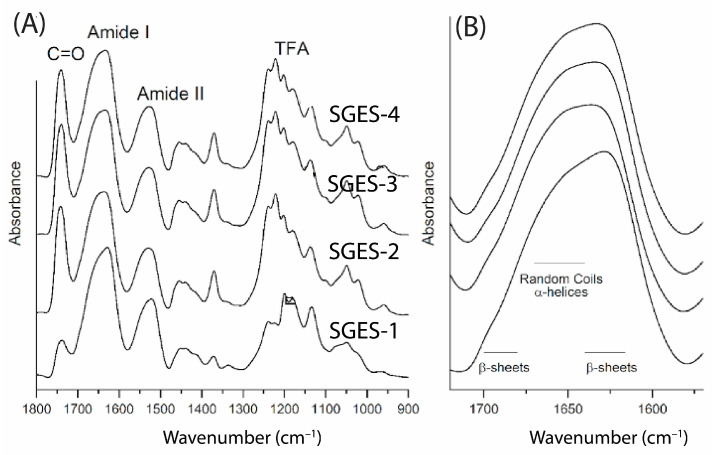
FTIR Spectra of Electrospun Sericin–Gelatin Mixtures. (**A**) ATR-FTIR absorption spectra in the 1800–900 cm^−1^ range for SGES-1, SGES-2, SGES-3, and SGES-4 samples, with the main peaks indicated. (**B**) Spectra are reported in the Amide I band.

The Sericin–Gelatin Electrospun Samples (SGES-1, SGES-2, SGES-3, and SGES-4) were analyzed using ATR-FTIR spectroscopy. These samples were electrospun under the following conditions: SGES-1 at a collector distance of 10 cm with a flow rate of 1 mL/h, SGES-2 at 15 cm with 0.8 mL/h, SGES-3 at 15 cm with 1 mL/h, and SGES-4 at 20 cm with 1 mL/h. The ATR-FTIR spectra of the samples obtained from the electrospinning of a sericin–gelatin mixture at a 1:1 ratio (Figure 4A) displayed the typical protein bands, including the Amide I and Amide II bands, as well as peaks associated with TFA. Specifically, the peak at 1740 cm^−1^ corresponds to C = O stretching, while peaks in the 1250–1100 cm^−1^ range are attributed to C–F vibrations. The relative intensities of these peaks (Figure S2) suggest the presence of residual TFA in varying amounts, with SGES-1 containing a lower amount compared to SGES-2 through SGES-4 and SGES-3.

Despite being electrospun at the shortest collector distance (10 cm), which should have limited in-flight solvent evaporation, SGES-1 retained the least residual TFA. This can be attributed to its more open fiber structure, as seen in SEM images in Section 2.3.2, which likely facilitated better post-deposition solvent removal. By contrast, SGES-2 to SGES-4 and SGES-3 exhibited denser fiber networks, suggesting that fiber morphology influenced solvent retention.

The Amide I band (Figure 4B) of the SGES samples revealed the presence of β-sheets and disordered structures. The SGES-1 sample exhibited a β-sheet content of 33%, which decreased to approximately 23% in the SGES-2 sample and to 27% in the SGES-3 and SGES-4 samples (Figure S3).

These findings highlight the importance of both secondary structures for fiber formation: the β-sheets contribute to inter-protein interactions, providing structural stability, while the disordered structures allow flexibility of the polypeptide chain, enabling its accommodation within the fiber.

### 2.3. Morphological Properties of Electrospun Samples

#### 2.3.1. Morphological Properties of Sericin Electrospun Samples

The morphology of electrospun sericin samples was analyzed using SEM micrographs to examine the effect of solution concentration on fiber formation. Sericin solutions ranging from 8 wt% to 12 wt% were electrospun.

**Figure 5:**
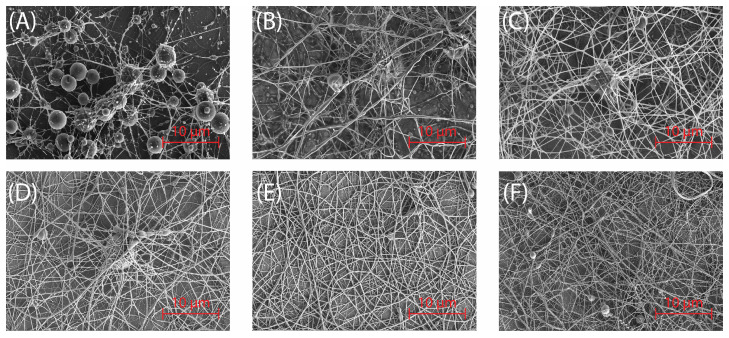
SEM images of electrospun sericin fibers prepared at different concentrations of sericin solution: (**A**) 8 wt%, (**B**) 9 wt%, (**C**) 10 wt%, (**D**) 12 wt% at 10 cm collector distance, (**E**) 12 wt% at 15 cm collector distance, and (**F**) 12 wt% at 20 cm collector distance.

In Figure 5A, corresponding to 8 wt% sericin solution, the network is dominated by bead-like structures connected by thin fibers. Large gaps and irregular distributions suggest suboptimal solution properties, preventing uniform fiber formation. Figure 5B, prepared with 9 wt%, shows fewer beads compared to the 8 wt% sample, with the presence of spindle-like beads and beaded fibers. Figure 5C, at 10 wt%, demonstrates further improvement, with smoother and thicker fibers, fewer beads, and a denser network.

At 12 wt%, the morphology improves significantly, with predominantly smooth, continuous fibers and minimal bead formation. Figure 5D–F correspond to the SES-1, SES-2, and SES-3 samples prepared as described in Section 2.2.2. This concentration was optimal for fiber formation, allowing for the analysis of fiber diameters across the different collector distances. Solution concentration proved to be the key factor, with higher viscosity at elevated concentrations yielding smooth, uniform fibers, while lower concentrations resulted in suboptimal morphologies [16]. The average diameters of the sericin nanofibers were 595 nm, 361 nm, and 347 nm for collector distances of 10 cm, 15 cm, and 20 cm, respectively. These results indicate that the average fiber diameter increases as the collector distance decreases [17].

**Figure 6:**
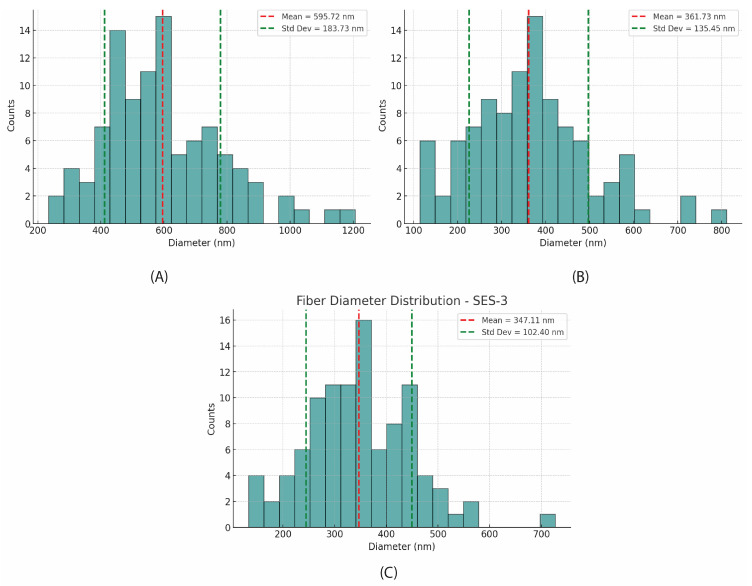
Fiber diameter distribution of electrospun sericin nanofibers at different collector distances. (**A**) SES-1 at 10 cm, (**B**) SES-2 at 15 cm, (**C**) SES-3 at 20 cm.

The distributions for SES-2 and SES-3, shown in Figure 6B,C, exhibit Gaussian-like profiles, indicating more uniform fiber production. SES-1 (Figure 6A), however, displays a broader distribution, with a higher standard deviation of 183.73 nm, suggesting greater variability in fiber diameters. The broader distribution observed for SES-1 may be attributed to factors such as higher stretching forces or environmental influences during electrospinning.

#### 2.3.2. Morphological Properties of Sericin–Gelatin Electrospun Samples

The analysis was conducted on 12 wt% sericin–gelatin solutions, previously identified as the optimal concentration for fiber formation (Section 4.6.2). Solutions with sericin-to-gelatin ratios of 1:2 and 2:1 exhibited excessive viscosity, leading to fiber bundling, with multiple fibers clustered together instead of forming uniform structures. This instability in the electrospinning jet caused irregular fiber morphologies. Consequently, the 1:1 sericin-to-gelatin ratio at 12 wt% (SGES-1 to SGES-4) was selected for further analysis due to its balanced viscosity and improved electrospinning performance.

**Figure 7:**
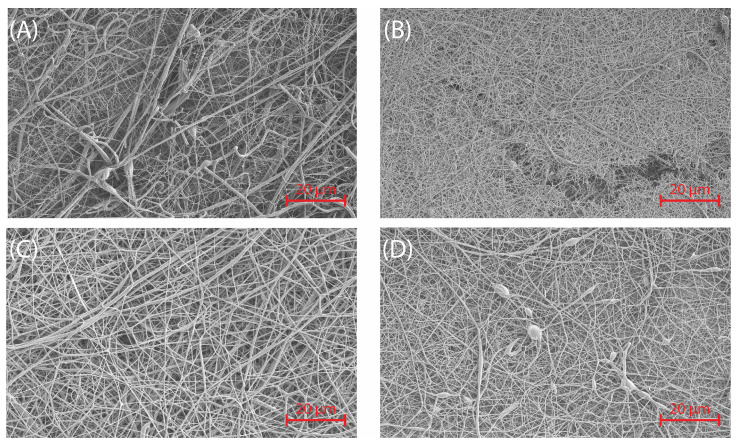
SEM images of electrospun sericin–gelatin fibers prepared at a constant concentration of 12 wt% with varying collector distances and flow rates: (**A**) 10 cm at 1 mL/h, (**B**) 15 cm at 0.8 mL/h, (**C**) 15 cm at 1 mL/h, and (**D**) 20 cm at 1 mL/h.

**Figure 8:**
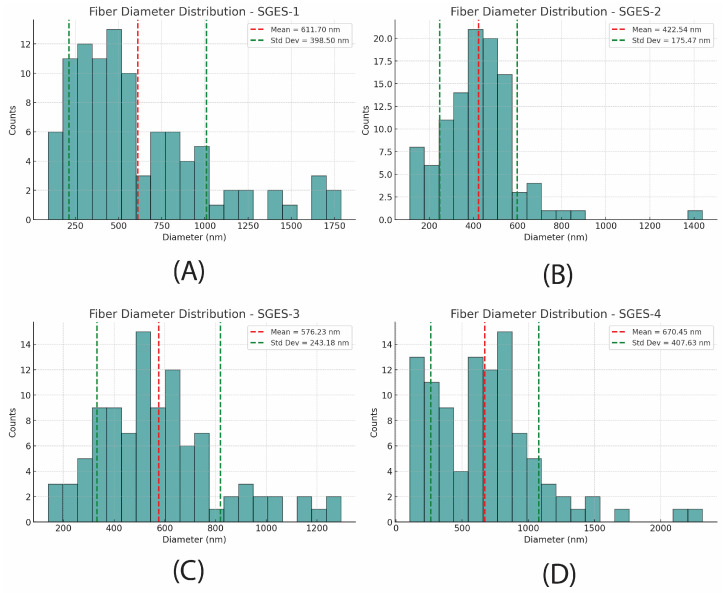
Fiber diameter distribution of electrospun sericin–gelatin fibers prepared at a constant concentration of 12 wt% with varying collector distances and flow rates: (**A**) 10 cm at 1 mL/h, (**B**) 15 cm at 0.8 mL/h, (**C**) 15 cm at 1 mL/h, and (**D**) 20 cm at 1 mL/h.

The fiber diameter distributions of SGES samples show varying degrees of uniformity. SEM images (Figure 7) and histograms (Figure 8) illustrate these differences.

SGES-1 (Figure 7A) exhibits significant thickness variations, with a broad and skewed fiber diameter distribution (Figure 8A) primarily concentrated between 200 and 600 nm. The presence of larger fibers above 900 nm contributes to the high standard deviation, likely due to the short 10 cm collector-needle distance, which limited fiber stretching and increased deposition variability.

A more uniform fiber structure is observed in SGES-2 (Figure 7B), where reduced beading and a moderately skewed distribution (Figure 8B) centered around 200–500 nm indicate improved fiber diameter uniformity. The increased 15 cm collector-needle distance and lower 0.8 mL/h flow rate helped refine fiber uniformity. However, the reduced flow rate introduced challenges, including droplet formation at the needle tip and occasional fiber breakage.

Among all samples, SGES-3 (Figure 7C) exhibits the most uniform fiber distribution, forming a Gaussian-like curve (400–700 nm) with minimal outliers (Figure 8C). With the collector distance maintained at 15 cm but the flow rate increased to 1 mL/h, the resulting fibers are thicker (average diameter: 576 nm), likely due to reduced jet stretching efficiency. Despite this, they maintain a consistent morphology and a relatively uniform structure, showing no signs of process instability. These conditions provided the best balance between fiber stretching and deposition stability, yielding the most uniform results.

A broader, slightly skewed distribution (600–900 nm) is observed in SGES-4 (Figure 7D), which exhibits the largest average fiber diameter (670 nm, Figure 8D). The 20 cm collector distance facilitated greater fiber stretching, but morphological inconsistencies emerged, including occasional beaded fibers. These defects suggest an interplay between reduced jet stability and incomplete solvent evaporation.

A lower flow rate (SGES-2) improves uniformity, while SGES-3 demonstrates that an intermediate distance with an optimized flow rate achieves the best balance of uniformity and stability. These findings underscore the importance of parameter fine-tuning for consistent fiber formation.

**Figure 9:**
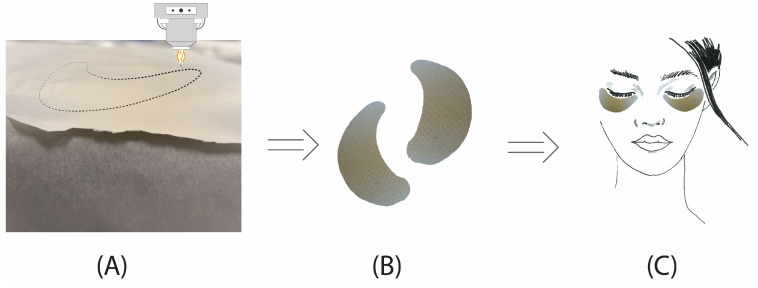
(**A**) Sericin–gelatin electrospun film being laser-cut into an eye contour shape. (**B**) Laser-cut eye contour patches prepared from sericin–gelatin electrospun films. (**C**) Application of the eye contour patches on a model’s face.

Following the characterization of electrospun films, the SGES-3 electrospinning parameters were identified as the most suitable for the development of eye patches, as they produced films with balanced mechanical integrity, homogeneity, and ease of handling. Using these parameters, an 8 mL solution was electrospun over a 2-h period to create films with uniform morphology. The films were allowed to dry in a fume hood at room temperature (22–25 °C) for 24 h, allowing residual TFA to evaporate before use. The drying process took place under standard laboratory humidity conditions (~45–50% RH). After drying, the films were laser-cut into the desired shape of eye patches and stored in a desiccator to prevent moisture absorption and preserve their structural integrity. Figure 9 shows the final prepared patches, demonstrating their suitability for targeted eye contour treatments.

**Figure 10:**
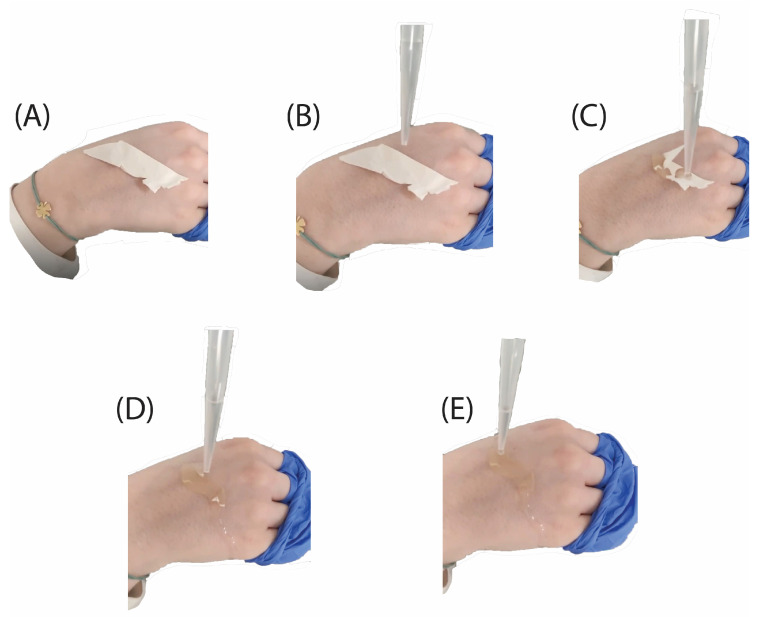
Sequential absorption and integration of the sericin–gelatin film upon water application. (**A**) The SGES film is applied to the skin of the hand. (**B**) Water is introduced to the surface of the SGES film. (**C**) Upon initial contact, the film begins to absorb water, initiating its integration into the skin. (**D**) Additional water is applied, facilitating further absorption and enhancing the interaction between the film and the skin. (**E**) The film demonstrates partial absorption into the skin.

Notably, the prepared eye patches exhibited water-soluble properties, partially dissolving upon contact with skin and water during application, as can be seen in Figure 10 below. This characteristic, attributed to the hydrophilic nature of sericin, enhances the interaction of the patches with the skin, facilitating their adherence and potential release of active components during use [1].

### 2.4. Cosmetic Performance of Sericin–Gelatin Electrospun Films

#### 2.4.1. Effect of Electrospun Films on Skin Hydration

A statistically significant decrease in the mean basal value of skin hydration was observed 30 min after product application (*p* < 0.0001). Table 2 displays the mean values, standard deviations, and statistical variations at the two time points: baseline (T_0_) and 30 min post-application (T_30__min_).

**Figure 11:**
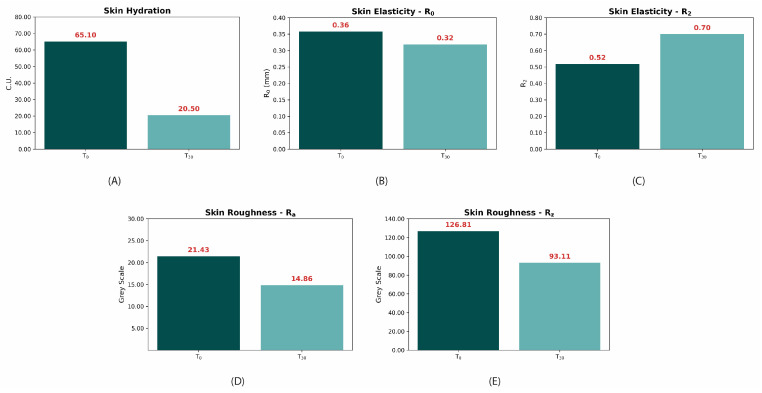
Comparative analysis of: (**A**) skin hydration mean values at baseline (T_0_) and 30 min (T_30__min_) post-application, (**B**) R_0_ parameter values at baseline (T_0_) and after 30 min (T_30__min_), (**C**) R_2_ parameter values at baseline (T_0_) and after 30 min (T_30__min_), (**D**) R_a_ parameter values at baseline (T_0_) and after 30 min (T_30__min_), and (**E**) R_z_ parameter values at baseline (T_0_) and after 30 min (T_30__min_).

At baseline (T_0_), the skin hydration value was 65.1 ± 14.2 C.U., whereas at 30 min (T_30min_) the value decreased to 20.5 ± 11.9 C.U., representing a relative reduction of –68.5%. This significant reduction is further illustrated in Figure 11A, which highlights the decline in skin hydration values over time.

The observed reduction confirms the effect of the tested product, as evidenced by the statistical test result (*p* < 0.0001), demonstrating a marked and reproducible loss of skin hydration within the 30-min time frame.

#### 2.4.2. Impact of Electrospun Films on Skin Elasticity

In terms of skin elasticity, changes in the R₀ parameter (skin extensibility) were not statistically significant (*p* > 0.05), despite a decrease from 0.358 ± 0.060 mm at baseline (T_0_) to 0.319 ± 0.107 mm at 30 min (T_30__min_), representing a −10.9% reduction (Table 2, Figure 11B).

Conversely, a significant improvement was noted in the R_2_ parameter (gross elasticity), with values increasing from 0.518 ± 0.067 at baseline to 0.700 ± 0.192 after 30 min (*p* < 0.01), reflecting a +35.1% improvement (Table 2, Figure 11C). This result underscores the product’s efficacy in enhancing gross skin elasticity.

#### 2.4.3. Reduction of Skin Roughness by Electrospun Films

The analysis of skin roughness revealed statistically significant reductions in both R_a_ (average roughness) and R_z_ (average maximum roughness) parameters.

The R_a_ parameter decreased from 21.43 ± 5.77 at baseline to 14.86 ± 3.06 at 30 min (−30.7%, *p* < 0.0001), as presented in Table 2 and illustrated in Figure 11D.

Similarly, the R_z_ parameter decreased significantly from 126.81 ± 25.58 to 93.11 ± 18.27 after 30 min, corresponding to a relative reduction of −26.6% (Table 2, Figure 11E).

These findings suggest a substantial improvement in skin smoothness and a reduction in surface irregularities following the application.

**Figure 12:**
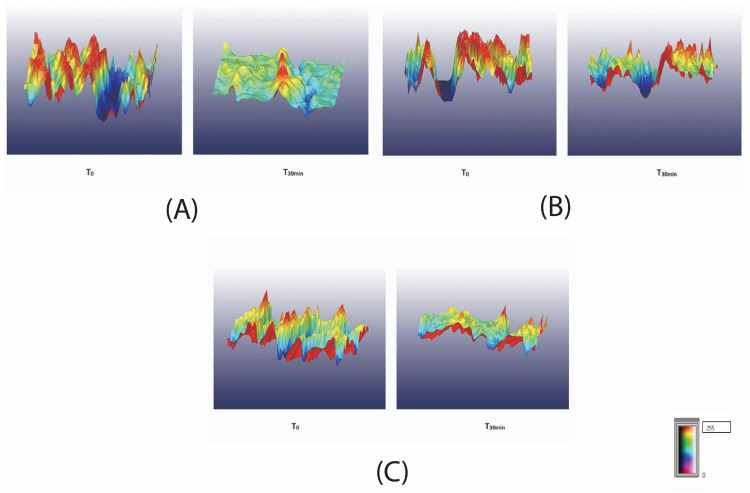
3D surface representation of skin roughness for: (**A**) subject 2 at baseline (T_0_) and 30 min (T_30__min_), (**B**) subject 4 at baseline (T_0_) and 30 min (T_30__min_), and (**C**) subject 17 at baseline (T_0_) and 30 min (T_30__min_).

To further analyze the changes in skin roughness, 3D surface representations were generated at baseline (T_0_) and 30 min (T_30min_) post-application. These visualizations (Figure 12), obtained from randomly selected subjects (2, 4, and 17), illustrate variations in roughness distribution, as indicated by changes in surface irregularities and color intensity across subjects. 

The colors in these images correspond to a predefined roughness scale, where different hues represent varying levels of surface texture. As shown in the legend within Figure 12, the scale ranges from 0 (smooth surface, blue regions) to 255 (highest roughness, red regions), providing a standardized visualization of roughness variations. This color mapping facilitates an objective assessment of changes in skin texture before and after treatment.

Table 3 provides an overview of the average roughness (R_a_) and average maximum roughness (R_z_) values at T_0_ and T_30__min_ for each subject, while Table 4 quantifies the absolute reduction in roughness (ΔR_a_ and ΔR_z_) to highlight differences in treatment effectiveness.

For Subject 2, the highest initial roughness was observed (R_a_ = 34.36, R_z_ = 175.94), suggesting a more pronounced skin texture. After 30 min, the most substantial reduction in roughness was recorded (ΔR_a_ = 22.34, ΔR_z_ = 101.21), with R_a_ decreasing to 12.02 and R_z_ dropping to 74.43, indicating a significant smoothing effect. The 3D visualization (Figure 12A) further supports this finding, showing a reduction in peak prominence and an overall more uniform surface. The color distribution in the images aligns with these quantitative results, with a shift from dominant red and yellow areas (higher roughness) at T_0_ to more green and blue regions (smoother surface) at T_30__min_, illustrating the decrease in skin irregularities.

Subject 4 started with moderately high roughness values (R_a_ = 31.09, R_z_ = 168.34) and demonstrated a noticeable but slightly less pronounced improvement (ΔR_a_ = 12.75, ΔR_z_ = 50.23), with final values of R_a_ = 18.34 and R_z_ = 118.11. The 3D visualization (Figure 12B) reflects this reduction, showing softened peaks and shallower valleys, though residual roughness remains higher than that of Subject 2. The visual representation of roughness aligns with these findings, showing a transition from predominantly red and yellow regions (higher roughness) at T_0_ to a combination of green, blue, and residual red areas at T_30__min_, suggesting partial smoothing with some remaining rough zones.

Subject 17 had the lowest initial roughness (R_a_ = 25.91, R_z_ = 157.93) and exhibited the least reduction post-treatment (ΔR_a_ = 6.82, ΔR_z_ = 45.61), resulting in R_a_ = 19.09 and R_z_ = 112.12 at T_30__nin_. While improvement was observed, the overall smoothing effect was less pronounced compared to the other subjects, as reflected in the 3D visualization (Figure 12C), which highlights a reduction in high-roughness areas but a still relatively uneven surface. This improvement is also reflected in the color transitions, where the initially dominant red and yellow tones (high roughness) at T_0_ shift towards an increased presence of green and blue areas at T_30__min_, indicating a smoother skin surface with reduced irregularities.

Overall, the findings confirm the effectiveness of the treatment in reducing surface roughness across all subjects. However, differences in the extent of improvement suggest that baseline roughness, individual skin characteristics, and treatment absorption efficiency may have influenced the results. The data from Table 3 and Table 4 indicate that higher initial roughness (as seen in Subject 2) corresponds to a greater reduction, whereas subjects with lower baseline roughness (Subject 17) exhibit less dramatic improvements. This trend suggests that the treatment may have a more pronounced effect on skin with greater textural irregularities.

## 3. Discussion

Sericin proteins have been reported to exhibit molecular weights ranging from 10 to 400 kDa, depending on the extraction method and conditions [18,19]. In our study, Elution 1A exhibited prominent bands primarily between 10 and 37 kDa, indicating successful enrichment of sericin within this molecular weight range. Elution 1B displayed distinct bands up to 25 kDa, accompanied by a smeared band spanning the 25–40 kDa range, reflecting a broader molecular weight distribution.

These results align with the findings of Baht et al., who observed molecular weights between 15 and 25 kDa using autoclave extraction, and partially with Gulrajani et al., who identified higher molecular weight fractions (25, 66, and 90 kDa) with diffuse bands above 205 kDa using the optimized HTHP methods [20,21]. The absence of higher molecular weight fractions (>50 kDa) in our study may reflect the extended extraction time (60 min at 121 °C), which likely induced selective hydrolysis, enriching lower molecular weight fractions.

Accordingly, SEC analysis of the HTHP sample revealed sericin components at 23.8 kDa, 12.8 kDa, and 9.5 kDa. These molecular weights align with reported optimal ranges for cosmetic applications, particularly in enhancing hydration, elasticity, and smoothness of the skin [22,23]. Lower molecular weight fractions, such as those identified in this study, are advantageous due to their improved skin penetration and film-forming properties.

The secondary structure of sericin samples, including the HTHP sericin and electrospun films (SES and SGES), was analyzed using CD and ATR-FTIR. The HTHP sericin exhibited predominantly disordered structures, as shown by a broad Amide I band at 1644 cm^−1^, characteristic of random coil and α-helix structures, with minor β-sheet contributions [24,25,26].

Electrospinning led to an increase in β-sheet content, as indicated by Amide I peaks at 1621 cm^−1^ and 1698 cm^−1^ in ATR-FTIR spectra. This suggests an enhanced structural stability due to crystalline β-sheets, while the remaining random coils allow flexibility within the fiber matrix. The transformation likely results from mechanical stretching and rapid solvent evaporation [27,28]. Residual TFA in the fibers, detected by peaks at 1740 cm^−1^ and 1200–1135 cm^−1^, may interfere with hydrogen bonding and crystallization [29].

For pure sericin electrospun films, ATR-FTIR analysis revealed an increase in β-sheet content compared to the non-electrospun sericin sample (HTHP SP, 17%), reaching ~30% in the SES films. Residual TFA was detected in all SES films, with the highest relative intensities observed in SES-3, followed by SES-2 and SES-1. For sericin–gelatin electrospun films, the β-sheet content was highest in SGES-1 (33%), followed by SGES-3 and SGES-4 (27%), while SGES-2 exhibited the lowest content (23%)**.** Residual TFA was also detected in SGES samples, with SGES-1 containing a lower amount compared to SGES-2, SGES-3, and SGES-4. The observed variations in molecular organization suggest that electrospinning primarily drives β-sheet formation, with gelatin incorporation potentially modulating the secondary structure in the films.

The morphological analysis of electrospun sericin samples highlighted the influence of solution concentration and electrospinning parameters on fiber formation. While lower concentrations resulted in bead-dominated networks, the 12 wt% sericin solution produced smooth, continuous fibers with minimal bead formation. This finding aligns with previous studies emphasizing the critical role of viscosity in fiber formation [16]. However, the average fiber diameters obtained (595 nm at 10 cm, 361 nm at 15 cm, and 347 nm at 20 cm) were larger than the values typically reported in the literature, which are around 200 nm [27,28]. Factors contributing to these differences may include variations in solution properties, such as molecular weight and viscosity, environmental conditions during electrospinning, and differences in the equipment used.

The addition of gelatin to sericin solutions significantly improved the mechanical integrity of the electrospun films, addressing the limitations of sericin’s mechanical properties and enabling their application as eye patches. The SGES-3 parameters (15 cm collector distance, 1 mL/h flow rate) produced the most optimal results, yielding fibers with consistent morphology and relatively uniform diameters, averaging 576 nm. Notably, sericin contributed to this increase in fiber diameter, consistent with findings from a previous study where the addition of sericin to gelatin solutions resulted in larger fibers with wider and more irregular diameter distributions due to its impact on solution viscosity and jet dynamics [30].

The sericin–gelatin electrospun films demonstrated both beneficial and unexpected effects on skin properties. Despite sericin’s inherent hydrophilicity, the observed decrease in skin hydration suggests that additional factors influenced moisture retention. One potential contributor is the presence of residual TFA, as indicated by ATR-FTIR analysis. TFA residues may have disrupted lipid organization in the stratum corneum and lowered skin pH, potentially leading to increased transepidermal water loss (TEWL) [31,32]. Furthermore, both sericin and gelatin lack strong occlusive properties, meaning that while they can bind water, they do not create an effective barrier to prevent its evaporation. The structural characteristics of the electrospun film may have also played a role, as its initial adherence to the skin could have temporarily impeded moisture penetration, limiting hydration retention.

Conversely, the significant increase in gross elasticity (R_2_: +35.1%) suggests that the sericin–gelatin film influenced the skin’s biomechanical properties beyond simple moisture retention. Sericin has been reported to stimulate collagen synthesis, which may enhance extracellular matrix integrity and improve skin resilience over time [33]. Additionally, gelatin, a biopolymer derived from collagen, likely contributed to this effect by providing structural reinforcement [34,35]. Given that collagen production improves skin tensile strength and elasticity, this interaction may explain the observed increase in elasticity despite the decrease in hydration.

The marked reduction in skin roughness (R_a_: –30.7%, R_z_: –26.6%) further supports the film’s ability to improve skin texture. This effect can be attributed to sericin’s strong affinity for keratin, allowing it to form a thin protective layer that smooths the skin’s microtopography [8]. By binding to keratin, sericin may have enhanced corneocyte cohesion, reducing uneven desquamation and leading to a more uniform skin surface. Additionally, the mild mechanical tightening effect of the dried film could have compacted the stratum corneum, further refining skin texture. The removal process itself might have provided mild exfoliation, eliminating loosely arranged corneocytes and contributing to the measured improvement in roughness parameters.

In summary, while the patches enhanced elasticity and smoothness, the unexpected hydration decline highlights the need for further optimization. Future studies should focus on reducing residual TFA content and adjusting film composition to improve moisture retention while maintaining the beneficial effects on elasticity and smoothness.

## 4. Materials and Methods

### 4.1. Materials

Fresh *Bombyx mori* silk cocoons were generously provided by the Council of Research in Agriculture and Analysis of Agricultural Economics (https://www.crea.gov.it/, accessed on 12 December 2024). The cocoon preparation process included classification, cutting, pupae removal, and storage of the cocoon shells under controlled conditions to maintain their quality.

Porcine gelatin (Type A, powdered, gel strength ~300 g Bloom), suitable for electrophoresis and cell culture applications, was obtained from Sigma-Aldrich (St. Louis, MA, USA) Niacinamide and dialysis tubing with a molecular weight cut-off (MWCO) of 6–8 kDa were also sourced from Sigma-Aldrich. Trifluoroacetic acid (TFA, ≥99% purity) was procured from TCI Europe N.V. (Tokyo Chemical Industry, Zwijndrecht, Belgium).

### 4.2. Silk Sericin Extraction and Purification

Silk sericin was extracted from *Bombyx mori* cocoons using the HTHP method. Specifically, 15 g of *B. mori* silk cocoons were placed in a crystallizing dish, to which 600 mL of distilled water was added. The dish was then transferred to autoclave bins. The cocoons were autoclaved using an Asal Vapormatic 770 autoclave at 121 °C and 15 psi for 60 min.

The resulting aqueous solution was subsequently filtered through Fisherbrand™ Glass Funnel Filters with Sintered Glass Disc to remove silk fibroin and residual solids. The filtered solution was then dialyzed in cellulose dialysis tubing with a molecular weight cutoff (MWCO) of 6–8 kDa for 48 h against distilled water, with frequent exchanges of water. This dialysis step was performed to eliminate low-molecular-weight impurities and to concentrate the sericin, ensuring the protein’s high purity. The volume of the dialysis solution and the frequency of water changes (e.g., every 4–6 h) were carefully monitored to maintain constant osmotic conditions.

Following dialysis, the sericin solution was stored at –20 °C for subsequent analysis. Additionally, a portion of the solution was spray-dried using a Shanghai Pilotech YC-015 (Shanghai, China) spray dryer to obtain sericin powder, which was also employed for further characterization and application-specific testing. Spray drying was performed under specific parameters: inlet temperature of 120 °C and outlet temperature of 70 °C, with an airflow rate of 4.5 m^3^/h. The resulting sericin powder, hereafter referred to as HTHP SP, was stored in airtight containers at room temperature until use.

The pH of the sericin solution was measured and adjusted, if necessary, to maintain neutrality (~7.0), using 0.1 M NaOH or HCl before storage and analysis.

### 4.3. Molecular Weight Analysis of Silk Sericin by SDS-PAGE

The sericin solution obtained through the HTHP extraction method was quantified for protein content using a bicinchoninic acid (BCA) protein assay kit. A 50 mL aliquot of the solution from the 60-min autoclave extraction was treated with a protease inhibitor tablet. Two protein aliquots of 70 mg each were prepared. The first aliquot was filtered through Whatman #4 filter paper, with the waste fraction collected separately. The filtrate was solubilized in Laemmli buffer containing β-mercaptoethanol and heated at 99 °C to denature the proteins. This sample was further processed using ProteoMiner™ technology with 200 µL of beads, yielding two elutions (Elution 1A and Elution 1B), which were concentrated to 15 µL and mixed with Laemmli buffer. A similar protocol was applied to the second aliquot, utilizing 250 µL of beads. After incubation for 2 h and centrifugation at 4000 rpm for 10 min, the supernatant was discarded, and the pellet was washed with distilled water. Two additional elutions (Elution 2A and Elution 2B) were obtained, concentrated, and solubilized in Laemmli buffer.

SDS-PAGE was performed as described by Nicoletti et al. [36]. Gels consisted of a 4% polyacrylamide stacking layer (125 mM Tris–HCl, pH 6.8, 0.1% SDS) and a 12% resolving layer (375 mM Tris–HCl, pH 8.8, 0.1% SDS). Electrophoresis was conducted using a Tris-glycine buffer (pH 8.3) in the cathode and a Tris buffer (pH 8.8) in the anode. The process involved three voltage steps: 50 V for 20 min, 100 V for 40 min, and 150 V until the dye front reached the bottom of the gel. Staining was performed with Colloidal Coomassie Blue, and gels were destained using 7% (*v*/*v*) acetic acid in deionized water.

### 4.4. Protein Identification of Silk Sericin by Mass Spectrometry

Protein bands of interest were excised and washed with 50 mM ammonium bicarbonate (AmBic) and acetonitrile (ACN) at 56 °C under stirring to remove residual Coomassie stain. Reduction was performed using 1.5 mg/mL DTT in 50 mM AmBic at 56 °C, followed by alkylation with 10 mg/mL iodoacetamide in 50 mM AmBic at room temperature. Proteins were then digested overnight at 37 °C with 0.02 µg/µL trypsin in 25 mM AmBic. Peptides were purified and concentrated using Stage Tips packed with reverse-phase C18 material (Thermo Fisher Scientific, Norristown, PA, USA).

The peptide mixtures obtained from tryptic digestion of sericin were analyzed using an UltiMate™ 3000 RSLCnano system (Thermo Fisher Scientific) coupled with an LTQ XL ion trap mass spectrometer (Thermo Fisher Scientific). Prior to injection, 8 µL of the digested samples were cleaned and pre-concentrated on a reversed-phase trap column (Acclaim PepMap100, C18, 100 Å, 5 µm, 100 µm ID × 2 cm length, Thermo Fisher Scientific). After the clean-up step, the trap column was placed in series with a PicoFrit (Parkville, VIC, Australia) fused silica reverse-phase analytical column (C18 HALO, 90 Å, 2.7 µm, 75 µm ID × 10.5 cm length, New Objective, Littleton, MA, USA).

Peptide separation was performed using a nano-chromatography system operating at a constant flow rate of 300 mL/min. A gradient elution program was optimized for sericin peptides, starting with 4% buffer B (2% water and 0.1% formic acid in acetonitrile) and linearly increasing to 96% buffer B over 60 min, while buffer A (2% acetonitrile and 0.1% formic acid in water) was used as the mobile phase. The separated peptides were ionized using nano-ESI and introduced into the mass spectrometer, with data acquisition performed in positive ion mode. Full MS scans were recorded in the mass range of 350–1800 m/z, followed by MS/MS fragmentation of the five most intense ions using collision-induced dissociation (CID). To avoid redundant fragmentation, dynamically excluded ions were excluded from selection for 30 s.

Protein identification was conducted using the Mascot search engine (Version 2.3.01) through the Proteome Discoverer software (v. 1.2.0, Thermo Fisher Scientific). A specific database containing sericin and related proteins from *Bombyx mori* (UniProtKB/Swiss-Prot) was used for peptide matching. Search parameters included trypsin specificity with up to two missed cleavages allowed, carbamidomethylation of cysteine as a fixed modification, oxidation of methionine as a variable modification, and a false discovery rate (FDR) of 1% at the peptide level. The combination of high-resolution chromatography and sensitive mass spectrometry allowed precise identification of sericin peptides, confirming its primary and secondary structure characteristics.

### 4.5. Structural Characterization of HTHP Sericin Powder and Electrospun Films Using SEC, ATR-FTIR, and CD Spectroscopy

#### 4.5.1. Size-Exclusion Chromatography

Sericin powder (2 mg), obtained by the HTHP method, was resuspended in 1 mL of PBS. The sample was centrifuged at 10,000× *g* for 15 min, and the clarified supernatant was analyzed using an NGC Quest 10 Plus chromatography system (Bio-Rad, Hercules, CA, USA) equipped with a Superdex 10/200 column (GE Healthcare, Little Chalfont, UK), with a molecular weight cutoff range of 10–600 kDa. Chromatographic separation was carried out in PBS as the mobile phase at a flow rate of 0.8 mL min^−1^, with detection at 280 nm.

A calibration curve was constructed using molecular weight (MW) standards: ribonuclease A (13.7 kDa), ovalbumin (43.0 kDa), aldolase (158.0 kDa), ferritin (440.0 kDa), and thyroglobulin (669.0 kDa), as described in the Supplementary Materials. Briefly, for each MW standard the distribution coefficient (K_D_) was calculated as follows:

$$K_{D}=\frac{V_{E}-V_{0}}{V_{C}-V_{0}}$$

where *V_E_* is the elution volume, *V*_0_ the void volume determined using blue dextran (2000 kDa), and *V_C_* the column volume determined using uracil (0.112 kDa). The calibration curve Log(MW) vs K_D_ reported in Figure S1 (Supplementary Material) was linearly interpolated using OriginPro 2023 (Version 10.0.0.154, OriginLab Corporation, Northampton, MO, USA). Multi-Gaussian fitting of the SEC chromatogram was also performed using the same software. The eluted SEC fractions (1 mL) at retention volumes of 17 mL, 19 mL, and 20 mL were collected for further characterization by ATR-FTIR and CD spectroscopy.

#### 4.5.2. ATR-FTIR Spectroscopy

ATR-FTIR spectroscopy was employed to assess the preservation of protein secondary structures in sericin, following previously described methods [37,38]. Sericin exhibits characteristic amide bands, including Amide I (1700–1600 cm^−1^), Amide II (1600–1520 cm^−1^), and Amide III (1350–1200 cm^−1^), which reflect specific protein secondary structures [16,39,40,41].

For solid samples, such as sericin powder or electrospun films, the materials were placed in direct contact with the diamond element of an ATR device (Quest, Specac Ltd., Pleasantville, NY, USA). For solution-based samples, such as SEC fractions, 2 μL of the sample were deposited on the ATR crystal, and spectra were collected after water evaporation to form a protein film. ATR-FTIR spectra were recorded at room temperature using a Varian 670-IR spectrometer (Varian Australia Pty Ltd., Mulgrave, VIC, Australia) equipped with a nitrogen-cooled mercury cadmium telluride (MCT) detector. Measurement parameters included the following: 2 cm^−1^ spectral resolution, 25 kHz scan speed, 1024 coadded scans, and triangular apodization.

To resolve secondary structure components in the Amide I region, second-derivative spectra were calculated after smoothing the absorption spectra using the Savitzky–Golay method. FTIR data acquisition and processing were performed using Resolutions Pro software (Version 5.4.1.3412, Varian Australia Pty Ltd., Mulgrave, VIC, Australia).

#### 4.5.3. Circular Dichroism Spectroscopy

CD spectroscopy was utilized as a complementary technique to ATR-FTIR to further investigate sericin’s secondary structures. Sericin powder and SEC fractions were analyzed, with SEC samples resuspended in water. Spectra were recorded at room temperature using a Jasco J-815 spectropolarimeter (Jasco Corp., Tokyo, Japan). Measurements were carried out in a quartz cell with a 0.1 cm path length, over the spectral range of 250–195 nm, and averaged over three scans. Bandwidth and time response settings were 2 nm and 2 s, respectively. CD spectra were smoothed using the Savitzky–Golay method [38,42].

These combined SEC, ATR-FTIR, and CD spectroscopy approaches enabled a comprehensive characterization of sericin’s structural properties, including its molecular weight distribution, secondary structure composition, and processing-dependent conformational changes.

### 4.6. Electrospinning and Solution Preparation

#### 4.6.1. Optimization of Electrospinning Parameters for Sericin Nanofibers

The electrospinning process is strongly influenced by polymer solution concentration, which governs viscosity, viscoelastic properties, and surface tension—key factors affecting jet stability, fiber morphology, and overall fiber formation feasibility [39,40,41]. Optimizing these parameters is crucial for ensuring uniform nanofiber formation.

Previous studies have explored a broad concentration range in sericin electrospinning. Zhang et al. reported bead formation below 6 wt%, while 6–8 wt% enabled uniform nanofiber production [27]. Khan et al. observed that concentrations below 8.5 wt% primarily formed beads, while 9.6–16.5 wt% led to beaded fibers, and uniform, bead-free nanofibers were obtained only above 20.9 wt% [28]. These differences are likely due to sericin molecular weight, extraction methods, and solvent interactions, all of which influence solution viscosity and chain entanglement.

Building upon these insights, sericin was dissolved in TFA and stirred for 5 h at 22–25 °C to prepare solutions ranging from 2 wt% to 15 wt%. Solutions below 8 wt% resulted in electrospraying, while those exceeding 12 wt% exhibited excessive viscosity, rendering the solution too viscous for electrospinning. Consequently, the working concentration range was established between 8 wt% and 12 wt%.

To confirm that the selected concentration range was suitable for electrospinning, viscosity measurements were performed on solutions between 8 wt% and 12 wt% using a rotational rheometer with a cone-plate geometry (50 mm diameter, 1° angle) at 25 °C. The recorded viscosity values ranged from 4.5 to 14.2 Pa·s, aligning with the reported optimal range for stable fiber formation (1–20 Pa·s) [43,44].

The prepared solutions were then electrospun using a standard electrospinning setup (SKE Research Equipment, E-Fiber EF300). For each sericin concentration, three collector distances were tested: 10 cm, 15 cm, and 20 cm, with corresponding voltage settings of 17 kV, 25 kV, and 32–35 kV, respectively. Voltage was adjusted for each distance to maintain a constant electric field, ensuring consistent stretching forces on the polymer jet across all parameter combinations.

To minimize variations in fiber morphology, temperature and relative humidity were maintained at 22–25 °C and 45–50%, respectively. The sericin solution was loaded into a 10 mL polypropylene/polyethylene syringe (Sigma-Aldrich) fitted with a 21-gauge stainless steel needle and connected to the high-voltage power supply. A constant flow rate of 1 mL/h was controlled using a syringe pump for all solutions.

A grounded aluminum foil collector was positioned at the specified distances from the needle tip. Electrospinning was performed for 30 min per parameter combination, and the collected nanofibers were stored in a desiccator to prevent moisture absorption prior to analysis.

#### 4.6.2. Preparation of Sericin–Gelatin–Niacinamide Solutions

To develop optimized nanofiber formulations, sericin–gelatin solutions at a total concentration of 12 wt% were prepared by dissolving sericin and gelatin in TFA at varying weight ratios (1:1, 1:2, and 2:1). The concentration of 12 wt% was selected based on previous electrospinning experiments with sericin, where it provided the most consistent fiber formation and optimal morphological results. Niacinamide was incorporated into the solutions at a final concentration of 2 wt% to enhance the potential bioactivity of the resulting nanofibers [45]. The solutions were stirred for 8 h at room temperature (22–25 °C) to ensure complete dissolution and homogeneity.

Electrospinning was performed under the same setup and environmental conditions described in Section 4.6.1, with flow rates ranging from 0.8 to 1 mL/h, collector distances of 10, 15, and 20 cm, and applied voltages of 17 kV, 25 kV, and 32–35 kV.

### 4.7. Morphological Characterization of Electrospun Films by Scanning Electron Microscopy (SEM)

Electrospun films were placed on adhesive carbon pads and chrome-coated with a thin layer of about 20 nm to enhance conductivity. SEM observations were conducted at the Platform of Microscopy of the University of Milano-Bicocca using a Zeiss Gemini 500 microscope.

Images were acquired under high vacuum at an accelerating voltage of 5 kV to analyze the surface morphology and fiber diameter. The mean diameter of the nanofibers was determined using the commercial software ImageJ (Version ImageJ 1.54g, National Institutes of Health, Bethesda, MD, USA), based on measurements of 100 randomly selected nanofibers from SEM images.

### 4.8. Cosmetic Evaluation of Electrospun Films

#### 4.8.1. Methodology and Statistical Analysis

Instrumental measurements were conducted in a temperature-controlled environment (24 ± 2 °C) to ensure consistency and minimize environmental variability during testing. This study involved 20 female volunteers, recruited to evaluate the effects of the electrospun films on skin hydration, elasticity, and roughness. Inclusion criteria ensured that participants had healthy skin in the tested area, and any skin conditions or recent cosmetic procedures were criteria for exclusion to maintain uniformity in the results.

Prior to the instrumental evaluations, participants were instructed to adhere to standardized preparation protocols. These included refraining from washing their faces for at least 2 h before the measurements and avoiding the application of any cosmetic products for a minimum of 12 h prior to the baseline assessment (T_0_).

The electrospun film applied to the eye contour area was SGES-3, selected for its mechanical integrity, homogeneity, and ease of handling (see Section 2.3.2, Figure 9). Prior to application, the eye contour area was lightly moistened to enhance adhesion and facilitate the dissolution of the water-soluble film. The patches, cut to 5.5 cm × 2.5 cm, were positioned to conform to the skin’s natural curvature, ensuring uniform contact and optimal performance.

Skin hydration, elasticity, and roughness were assessed at two specific time points: baseline (T_0_) and 30 min post-application (T_30min_). The measurements were performed using specialized instruments tailored to each parameter:

- Hydration: Assessed with the Corneometer® CM 825 to quantify water content in the stratum corneum.
- Elasticity: Evaluated using the Cutometer® MPA 580 to measure biomechanical properties of the skin.
- Roughness: Analyzed with skin replicas processed by Quantilines software (Version 1.1.7.0, Monaderm) to calculate surface texture parameters (R_a_ and R_z_).

The instrumental evaluations provided quantitative data to determine the effects of the electrospun films on the skin’s physical characteristics. Changes in hydration, elasticity, and surface roughness were calculated as differences between T_0_ and T_30min_, with improvements reflected in increased hydration and elasticity values, and decreased roughness parameters. These data offer insights into the potential cosmetic benefits of the electrospun films on skin health and appearance.

#### 4.8.2. Skin Hydration Analysis

The assessment of skin hydration levels was conducted using the Corneometer® CM 825, a widely utilized instrument in dermatological and cosmetic studies. The Corneometer operates on the principle of capacitance measurement, utilizing a square-shaped sensor with an area of 49 mm^2^, covered by a specialized glass and mounted on a movable axis. This configuration facilitates accurate measurements by ensuring stable sensor–skin contact.

When the sensor’s front surface was gently pressed against the skin, the instrument provided a numerical value on its liquid crystal display. This value, expressed in corneometric units (C.U.), was directly proportional to the water content in the stratum corneum, serving as a reliable indicator of skin hydration. The measurement range spanned from 0 to 150 C.U., with higher values reflecting increased hydration levels.

To minimize variability of the results, measurements were performed on a flat and undisturbed area of the skin, with consistent pressure and minimal probe movement. Each measurement was conducted within a short duration to prevent potential occlusion effects caused by prolonged contact with the sensor. Three consecutive readings were taken for each test area, and the mean value was calculated to account for potential variability.

The effectiveness of the tested films in improving skin hydration was determined by monitoring changes in average corneometric values over time. A statistically significant increase in these values indicated the hydrating potential of the applied formulations.

#### 4.8.3. Skin Elasticity Testing

Skin elasticity was evaluated using the Cutometer® MPA 580, a widely recognized instrument for assessing the biomechanical properties of the skin. The device measures the vertical deformation of the skin under controlled suction and release, providing quantitative data on its elastic and viscous behavior.

During the assessment, the skin was subjected to a constant air depression of 350 mbar for a duration of 3 s using the probe’s opening, followed by a release phase of equal duration (3 s). This suction–release cycle was repeated three times on the same skin site to ensure accuracy and reproducibility of the measurements.

**Figure 13:**
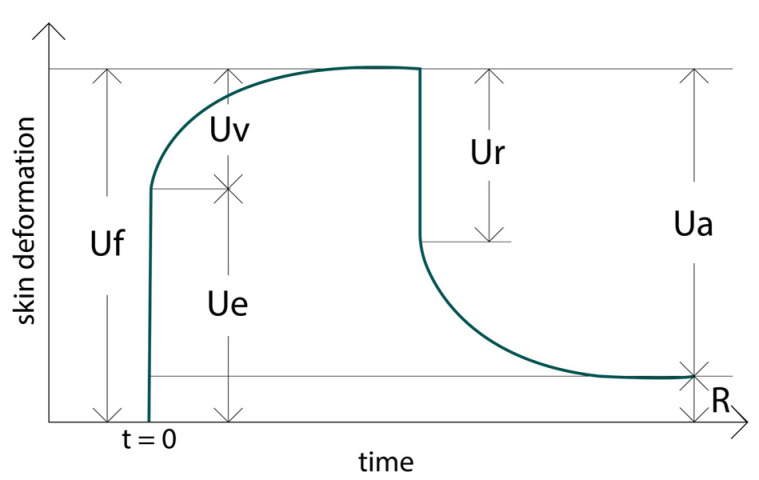
Representative Cutometer curve for skin deformation over time.

The deformation of the skin, expressed in millimeters (mm), was plotted as a function of time (in seconds), generating characteristic deformation curves for each cycle. Figure 13 illustrates a representative Cutometer curve, detailing the phases of skin deformation and recovery.

The graph displays the following components:

- UF (R_0_, Skin Extensibility): The initial deformation of the skin caused by the applied force, reflecting its passive behavior under stress.
- UE (Elastic Deformation): The portion of deformation that occurs immediately due to the application of stress.
- UV (Visco-Elastic Creep): The time-dependent deformation that continues to occur after the initial elastic deformation.
- UR (Immediate Recovery): The elastic recovery of the skin due to the removal of stress.
- UA (Total Recovery): The overall recovery at the end of the stress-off phase, quantifying the skin’s ability to return to its original position.
- R (Residual Deformation): The amount of deformation that is not recovered at the end of the stress-off time.

These components collectively represent the phases of skin deformation and recovery, with distinct contributions from elasticity and viscous behavior. In conclusion, the R_0_ parameter quantifies the skin’s extensibility, while the R_2_ parameter provides a comprehensive measure of gross elasticity. Together, these values offer valuable insights into the skin’s firmness, recovery potential, and overall mechanical properties.

#### 4.8.4. Skin Roughness Measurement

Skin roughness was evaluated using skin replicas and advanced image analysis techniques with specialized equipment and materials, including fast-hardening synthetic polymer (SILFLO, Monaderm, Monaco) and adhesive discs (3M, 24 × 40). A precise negative imprint of the skin surface was created using these materials.

The procedure began by applying an adhesive disc to the subject’s skin, ensuring a well-defined area for investigation while minimizing skin stretching during the polymer application. A small amount of the synthetic polymer was then carefully spread within the circular area defined by the adhesive disc. The polymer was left to dry until fully hardened, after which the disc was gently removed, leaving an accurate duplicate of the skin’s surface.

The obtained skin replicas were analyzed using the image processing software Quantilines (Version 1.1.7.0, Monaderm), enabling a comprehensive evaluation of the skin’s surface relief based on the method outlined by Corcuff [46]. To ensure precision in measurement, each silicon replica was illuminated by a light source at a defined incident angle (35°). This configuration accentuated surface features by creating shadows, where deeper furrows cast wider shadows. To optimize the analysis, the main wrinkles were oriented perpendicular to the direction of the incident light.

High-resolution images of the skin replicas, covering a surface area of 12 × 9 mm^2^, were captured using a video camera (Visioline^®^ VL650, Courage + Khazaka, Bucharest, Romania). The Quantilines software processed the images to calculate the following roughness parameters:

- R_a_ (average roughness): Represents the mean deviation of the surface profile from a reference line, providing an overall measure of the skin’s texture.
- R_z_ (average maximum roughness): Reflects the average difference between the highest and lowest points over five sections of the profile, indicating the depth of wrinkles and furrows.

Both R_a_ and R_z_ values were expressed in brightness units (grey levels), ranging from 0 to 255, enabling a standardized quantification of roughness. The analysis focused on the skin’s crow’s feet area, assessing the efficacy of the product in reducing roughness. A decrease in R_a_ and/or R_z_ values was interpreted as an improvement in skin smoothness and overall surface texture after treatment.

#### 4.8.5. Mathematical Elaboration

The instrumental data collected during this study were subjected to rigorous statistical analysis to evaluate the significance of changes in skin hydration, elasticity, and roughness following the application of the electrospun films. For each parameter, mean values, standard deviations, and variations were calculated to provide a comprehensive overview of the results.

To assess normality, the Kolmogorov–Smirnov test was applied. Comparisons between baseline (T_0_) and post-application (T_30min_) measurements were conducted using a paired *t*-test, with *p* < 0.05 considered statistically significant. This ensured robust evaluation of the product’s impact on skin hydration, elasticity, and roughness, offering meaningful insights into its cosmetic performance.

## Figures and Tables

**Table 1:** Molecular weight of HTHP sericin powder and its populations.

	Volume (mL)	MW (kDa)	Area (%)
HTHP SP	19.3	14.7	
HTHP SP-23	17.9	23.8	54.5
HTHP SP-12	19.7	12.8	34.5
HTHP SP-9	20.7	9.5	11.0

**Table 2:** Mean values, standard deviations, and statistical analyses of skin hydration and biomechanical parameters at baseline (T_0_) and 30 min (T_30__min_), including: (A) skin hydration, (B) R_0_ parameter (skin extensibility), (C) R_2_ parameter (gross elasticity), (D) R_a_ parameter (average roughness), and (E) R_z_ parameter (average maximum roughness).

	T_0_	T_30min_	T_30min_ − T_0_ (%)	*p* Level (T_0_ vs. T_30min_)
A	65.1± 14.2	20.5± 11.9	−44.6 (−68.5%)	*p* < 0.0001
B	0.358± 0.060	0.319± 0.107	−0.039 (−10.9%)	*p* > 0.05
C	0.518± 0.067	0.700± 0.192	+0.182 (+35.1%)	*p* < 0.01
D	21.43± 5.77	14.86± 3.06	−6.57 (−30.7%)	*p* < 0.0001
E	126.81± 25.68	93.11± 18.27	−33.70 (−26.6%)	*p* < 0.0001

**Table 3:** Surface Roughness Values (R_a_ and R_z_) at T_0_ and T_30min_ for each subject.

Subject	R_a_ (T_0_)	R_a_ (T_30min_)	R_z_ (T_0_)	R_z_ (T_30min_)
2	34.36	12.02	175.54	74.33
4	31.09	18.34	168.34	118.11
17	25.91	19.09	157.73	112.12

**Table 4:** Reduction in Surface Roughness (ΔR_a_ and ΔR_z_) between T_0_ and T_30min_ for each subject.

Subject	ΔR_a_ (T_0_ – T_30min_)	ΔR_z_ (T_0_ – T_30min_)
2	22.34	101.21
4	12.75	50.23
17	6.82	45.61

## Data Availability

The original contributions presented in this study are included in the article/Supplementary Materials. Further inquiries can be directed to the corresponding author.

## Supplementary Materials

The following supporting information can be downloaded at https://www.mdpi.com/article/10.3390/molecules30030715/s1. Figure S1: Size-exclusion chromatography calibration curve. The calibration curve was performed using a Superdex 10/200 column and the following MW standards: Ribonuclease A (RibA, MW: 13.7 kDa), Ovalbumin (Ova, MW: 43.0 kDa), Aldolase (Ald, MW: 158.0 kDa), Ferritin (Fer, MW: 440.0 kDa), Thyroglobulin (Thy, MW: 669.0 kDa); Figure S2: Ratios of the C=O and C-F peak areas to the Amide I (AI) band area reported for the different samples. While similar relative intensities were observed for the C=O/AI and C-F/AI ratios, the C=O/AI ratio provides a more accurate representation of the residual TFA content. This is because the C=O peak (centered at about 1740 cm^−1^) is not overlapped with other band components, unlike the two C-F peaks (the sum of the ~1200 cm^−1^ and of the ~1135 cm^−1^ band areas was considered), as shown in Figure 3 and Figure 4. Peak areas were obtained from the absorption spectra using the ResolutionPro software. Error bars represent the standard deviations from spectra collected in three different areas of protein films; Figure S3: Gaussian Curve Fitting and β-sheet content. (A) A representative curve fitting analysis is shown for each sample. The curve fitting was conducted in the 1750–1600 cm^−1^ spectral region [47] using the Orange software [48]. The ratio of the peaks assigned to β-sheets to the total Amide I band area is reported as the β-sheet percentage, as displayed in panel (B). Error bars represent the standard deviations from analyses performed on spectra collected in three different areas of the protein films.
