# Supplementary material for: Silk Sericin-Based Electrospun Nanofibers Forming Films for Cosmetic Applications: Preparation, Characterization, and Efficacy Evaluation
Source: Molecules. 2025 Feb 5;30(3):715. doi: 10.3390/molecules30030715 (PMC11820507; doi:10.3390/molecules30030715)
Supplement: Supplementary file 1 [file molecules-30-00715-s001.zip › molecules-3450501-supplementary.pdf]

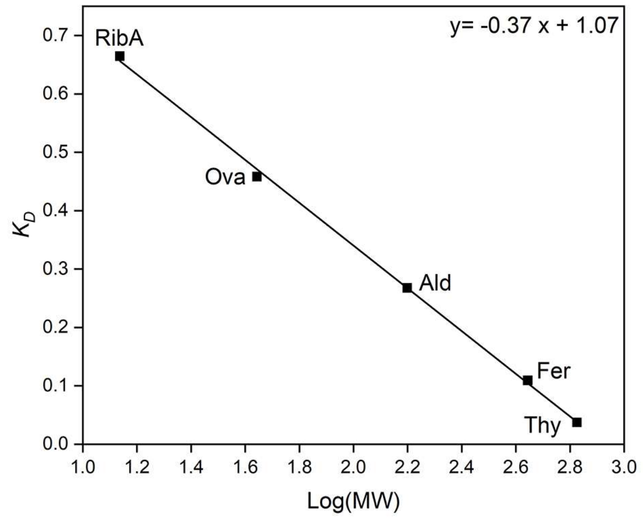

Figure S1. Size-exclusion chromatography calibration curve. The calibration curve was performed using a Superdex 10/200 column and the following MW standards: Ribonuclease A (RibA, MW: 13.7 kDa), Ovalbumin (Ova, MW: 43.0 kDa), Aldolase (Ald, MW: 158.0 kDa), Ferritin (Fer, MW: 440.0 kDa), Thyroglobulin (Thy, MW: 669.0 kDa).

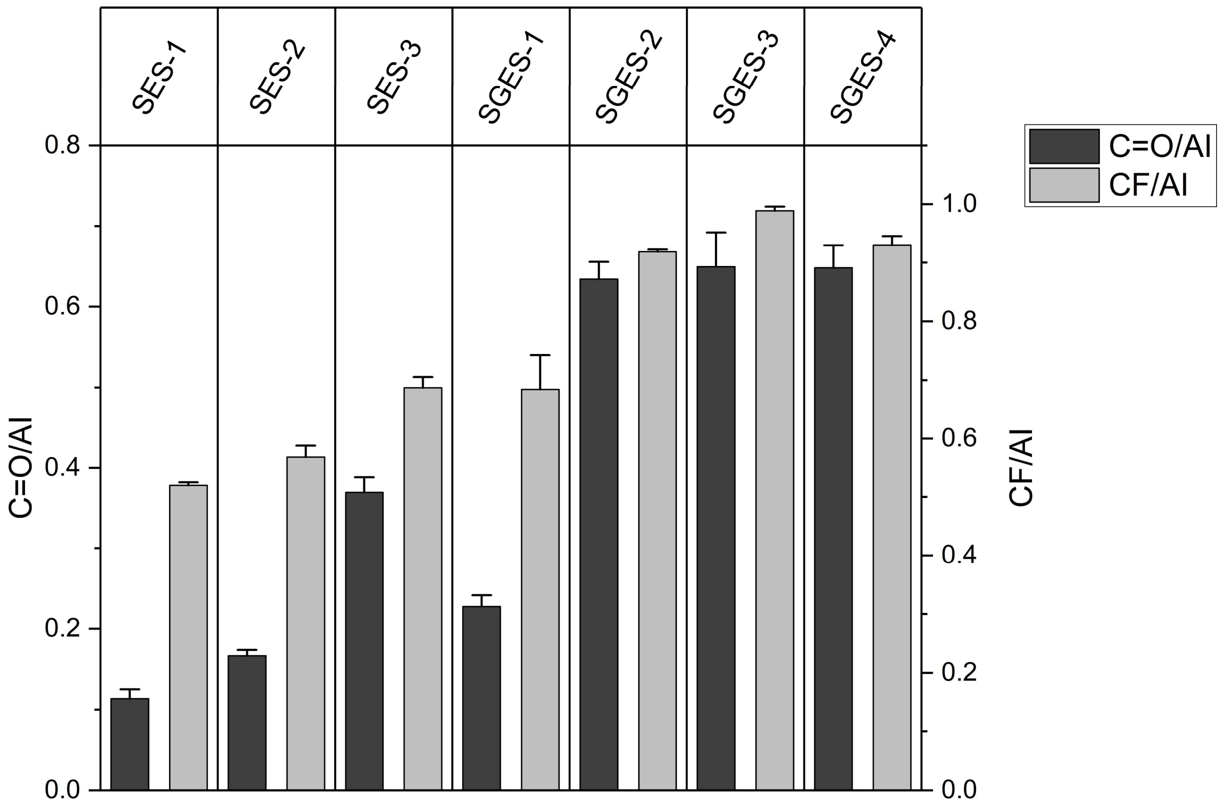

Figure S2. Ratios of the C=O and C-F peak areas to the Amide I (AI) band area reported for the different samples. While similar relative intensities were observed for the C=O/AI and C-F/AI ratios, the C=O/AI ratio provides a more accurate representation of the residual TFA content. This is because the C=O peak (centered at about 1740 cm^{-1}) is not overlapped with other band components, unlike the two C-F peaks (the sum of the ~1200 cm^{-1} and of the ~1135 cm^{-1} band areas was

considered), as shown in Figures 3 and 4. Peak areas were obtained from the absorption spectra using the ResolutionPro software. Error bars represent the standard deviations from spectra collected in three different areas of protein films.

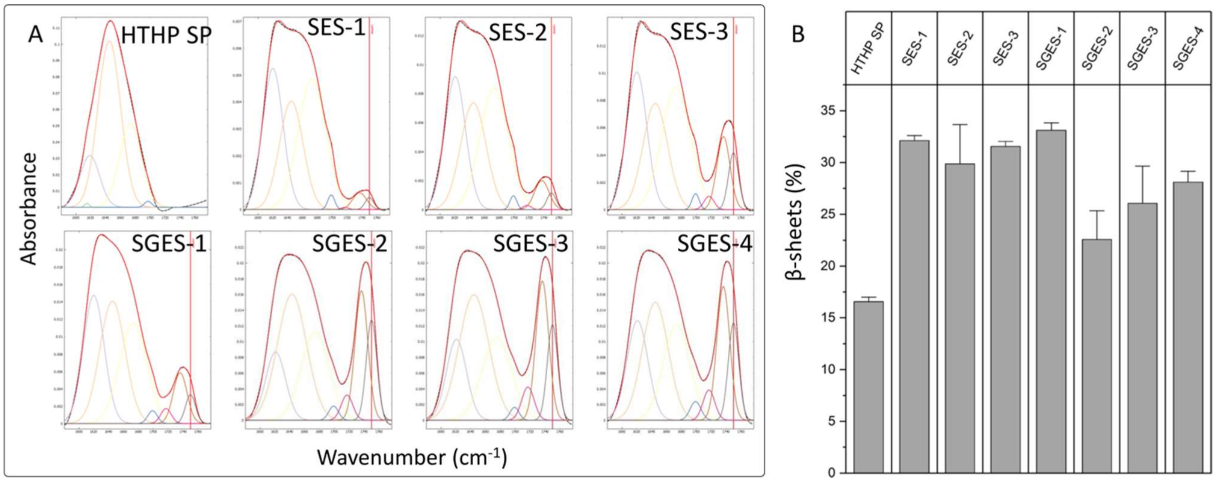

Figure S3. Gaussian Curve Fitting and β-sheet content. (A) A representative curve fitting analysis is shown for each sample. The curve fitting was conducted in the 1750–1600 cm^{-1} spectral region [1] using the Orange software [2]. The ratio of the peaks assigned to β-sheets to the total Amide I band area is reported as the β-sheet percentage, as displayed in panel (B). Error bars represent the standard deviations from analyses performed on spectra collected in three different areas of the protein films.

<u>Bibliography:</u>

1. Natalello, A.; Ami, D.; Brocca, S.; Lotti, M.; Doglia, S.M. Secondary Structure, Conformational Stability and Glycosylation of a Recombinant *Candida Rugosa* Lipase Studied by Fourier-Transform Infrared Spectroscopy. *Biochemical Journal* 2005, *385*, 511–517, doi:10.1042/BJ20041296.
2. Toplak, M.; Read, S.T.; Sandt, C.; Borondics, F. Quasar: Easy Machine Learning for Biospectroscopy. *Cells* 2021, *10*, 2300, doi:10.3390/cells10092300.
